# An Adaptive Learning Based Network Selection Approach for 5G Dynamic Environments †

**DOI:** 10.3390/e20040236

**Published:** 2018-03-29

**Authors:** Xiaohong Li, Ru Cao, Jianye Hao

**Affiliations:** 1 School of Computer Science and Technology, Tianjin University, Tianjin 300000, China; 2 School of Software, Tianjin University, Tianjin 300000, China

**Keywords:** network selection, dynamic bandwidth, reinforcement learning, prediction method

## Abstract

Networks will continue to become increasingly heterogeneous as we move toward 5G. Meanwhile, the intelligent programming of the core network makes the available radio resource be more changeable rather than static. In such a dynamic and heterogeneous network environment, how to help terminal users select optimal networks to access is challenging. Prior implementations of network selection are usually applicable for the environment with static radio resources, while they cannot handle the unpredictable dynamics in 5G network environments. To this end, this paper considers both the fluctuation of radio resources and the variation of user demand. We model the access network selection scenario as a multiagent coordination problem, in which a bunch of rationally terminal users compete to maximize their benefits with incomplete information about the environment (no prior knowledge of network resource and other users’ choices). Then, an adaptive learning based strategy is proposed, which enables users to adaptively adjust their selections in response to the gradually or abruptly changing environment. The system is experimentally shown to converge to Nash equilibrium, which also turns out to be both Pareto optimal and socially optimal. Extensive simulation results show that our approach achieves significantly better performance compared with two learning and non-learning based approaches in terms of load balancing, user payoff and the overall bandwidth utilization efficiency. In addition, the system has a good robustness performance under the condition with non-compliant terminal users.

## 1. Introduction

Two major characteristics of the fifth generation wireless network (5G) are heterogeneity and intelligentization. In terms of heterogeneity, wireless communications in 5G are making efforts to integrate various wireless access technologies into a heterogeneous network (HetNet) environment. Overlapping networks such as LTE, WLAN and WiMAX provide multiple access choices for terminal users [1]. In term of intelligentization, different from the present network scenarios, diverse networks (specifically, base stations) are managed by a single radio controller (SRC) to achieve an unified and dynamic scheduling of radio resources, instead of allocating fixed resource blocks to each network [2]. This centralized deployment is able to improve the resource utilization and reduce the energy consumption. However, in the meantime, it also introduces challenges. One of the difficulties lies in making the optimal choice for each user to fully utilize the dynamically changing radio resources in multi-user, multi-provider HetNet environments [3].

Many efforts have been made to help select appropriate networks from all available candidates to satisfy users’ specific requirements. Commercial solutions usually involve rudimentary static network selection policies (e.g., always select the WLAN, or always select the cheapest or fastest network) [4]. However, varying network characteristics and user preferences are omitted, which may often result in lower quality of service (QoS). Many traditional methods in research literature use multi-attribute decision making algorithms to evaluate and rank candidate networks in a preference order to guide the selection decision [5,6,7]. This may cause the congestion situation when most users connect to the same so-called “best” network. Game theory is widely employed to study the behaviour of selfish users selecting the best access network and analyze the existence of equilibrium [4,8]. Q-Learning based approaches are used to find the best strategy to maximize the reward function expressed in terms of different criteria, such as received signal strength, perceived bandwidth, prices and so on [9,10]. Xu et al. in [11] give the simple analysis of the application of four kinds of distributed learning algorithms in wireless networks.

Unfortunately, the above works suffer from the following two limitations: (1) requiring too much state information (the amount of bandwidth, the number of users, other users’ choices, etc.) as a prior, which is costly or impractical; (2) only focusing on static resources without considering the changing characteristics in intelligent wireless network environments. Existing network selection algorithms may work well with sufficient information when wireless resources are unchangeable; however, they are vulnerable to uncertainties with incomplete knowledge in dynamic network environments. In addition, the solutions of network resource management problems have already been proposed in recent literature, which capture the decision of users to select a wireless internet service provider that fulfills their demands. In [12], the combined problem of network provider selection and corresponding power allocation is treated using machine learning mechanisms. Tsiropoulou et al. in [13] propose a learning algorithm to achieve an energy efficient resource allocation. They all take the power/energy resource into consideration; however, in the next intelligent network environment, the resource of bandwidth should be firstly considered because the dynamic of bandwidth resource is designed for reducing the power consumption.

To address the above problems, an adaptive learning based network selection strategy is proposed in this paper. We are faced with two challenging issues. The first one originates from the consideration of both varying user demand and changing radio resource. Secondly, given a bunch of selfish users, how to achieve a load balance among a variety of networks is challenging. We modeled the problem as a multiagent coordination problem. Within a dynamic and heterogeneous network environment, a population of rational terminal users compete to select the optimal access networks to satisfy their diverse requirements with incomplete information (no prior knowledge of changing bandwidth and other users’ choices). Then, a learning based network selection strategy is designed, which enables users to receive a good reward and adaptively adjust their selections.

Simulation results show that the system guarantees convergence towards Nash equilibrium, which is proved to be Pareto optimal and socially optimal. Extensive results demonstrate that our algorithm significantly outperforms either the learning or non-learning based approach in terms of load balancing, user payoff and the overall bandwidth utilization efficiency. In addition, the system is examined to have a good robustness performance under up to 50% non-compliant terminal users.

The contribution of the paper mainly includes the following three parts:

- The heterogeneous network selection scenario is abstracted as a multiagent coordination problem, and a corresponding mathematical model is established. We analyzed the theoretical results of the model, i.e., the system guarantees convergence towards Nash equilibrium, which is proved to be Pareto optimal and socially optimal.
- The multiagent network selection strategy is proposed and appropriate algorithms are designed that enable users to adaptively adjust their selections in response to the gradually or abruptly changing environment.
- The performances of the approach are investigated under various conditions and parameters. Moreover, we compare our results with two existing approaches and get significantly better performances. Finally, the robustness of our proposed approach is examined, for which the system keeps desirable performances with non-compliant terminal users.

The remainder of the paper is organized as follows. We introduce the background in Section 2. In Section 3, we formulate the model of the multiagent network selection problem. Then, we describe our adaptive learning based network selection strategy. Section 4 presents the experimental results, makes comparisons with existing approaches and examines the robustness for the adaptive approach. Finally, we discuss the advantages and the limitations in Section 5 and conclude the paper in Section 6.

## 2. Background

### 2.1. Game Theory

Game theory is a mathematical tool used in understanding and modelling the strategic interactions between rational decision-makers [4]. The idea is that multiple players compete or cooperate with each other to achieve the maximization of their own benefits or the global utility. There are three important properties in game theory:

- The most commonly adopted solution concept in game theory is **Nash equilibrium** (NE). Under an NE, no player can benefit by unilaterally deviating from its current strategy.
- An outcome is **Pareto optimal** if there does not exist any other outcome under which no player’s payoff is decreased while at least one player’s payoff is strictly increased.
- **Socially optimal** outcomes refer to those outcomes under which the sum of all players’ payoffs are maximized [14].

Ilaria et al. in [15] employ a congestion game to model the interactions among terminal users, where users selfishly select the access network that minimizes their costs. A mathematical programming in this paper is proposed to find NE in the game. A similar game is also used in [16] to study the behaviour of selfish users selecting the best radio access technology (RAT). They prove that the strategy converges to NE and, under some conditions, the NE is also Pareto optimal. Monsef et al. in [17] formulate the problem as a non-cooperative game, and study various properties (NE and fairness) of distributed network selection with priority-based service. However, in a few works, the three properties are satisfied simultaneously under a certain condition as our work (concretely analyzed in a later section). Especially for social optimality, it is desirable in this problem, since it may indicate the maximal utilization of the overall performance of the HetNet environment.

### 2.2. Q-Learning

Q-learning is a well-studied class of algorithms that allow agents to determine the ideal behaviour within a specific context to maximize its performance through trial and error exploration in an unknown environment [18,19]. It works by learning *Q*-function, which characterizes relative utility of a particular action. An agent uses its experience to improve its function, bending new information into its prior experience according to a learning rate, so that the actions which yield high rewards are reinforced.

Q-Learning based methods are promising candidate solutions used in the network selection problem because it doesn’t require much prior information and the selection result can be learnt to be better. A channel selection and Routing approach is proposed in [20], which models the problem as a Marko decision process to design the method of learning the best resource allocation policies. Q-learning is used in [21] to maximize the global reward in network selection decisions. The Q-learning algorithm is also modeled in [22] to find the best strategy to maximise the reward function expressed in terms of call blocking and call dropping probabilities. To sum up, existing Q-learning based network selection approaches fail in designing reward functions to directly select networks, which usually results in lower performance of other aspects except for the defined reward. From a different perspective, our optimal decisions of network selection mainly depend on the prediction and learning of the state information. The Q-learning algorithm is used to evaluate the accuracy of prediction and guarantee the network-level performance (more details are in later sections).

### 2.3. Dynamic HetNet Environments

**Figure 1:**
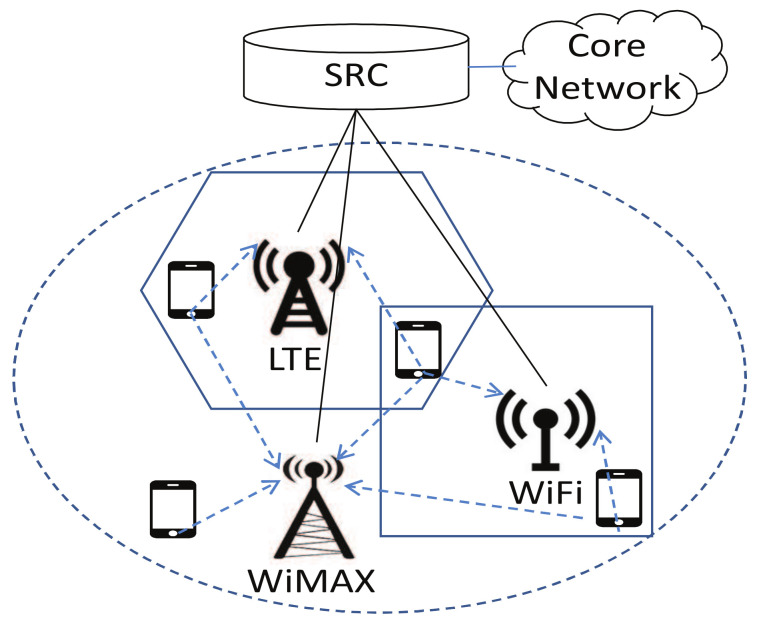
HetNet architecture in 5G—a sample secenario.

One emerging trend of the future 5G network is the increasing heterogeneity. A key feature therein would be the increasing integration between networks with different RATs [2]. Meanwhile, the core network will also reach unprecedented levels of flexibility and intelligence. Multiple base stations belonging to various networks are managed by a single radio controller (SRC) [23]. Under such a centralized deployment of HetNet architecture, radio resources are allocated dynamically by SRC to base stations as Figure 1. One practical example is the ZTE’s soft base station system [24]. Based on the emerging software defined radio (SDR) technology, the wireless resource of the soft base station in the system is capable of software programming and redefining.

**Figure 2:**
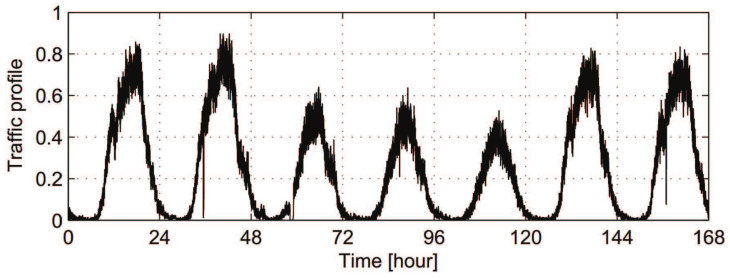
Normalized traffic profile during one week [25].

One natural way of dynamically allocating radio resources to different base stations is based on their changeable traffic conditions. Figure 2 shows one typical traffic profile in real cellular-based wireless access networks [25]. The traffic profile during the daytime period has higher value than that of the nighttime period. Since traditional base stations are planned to support the daytime traffic, infrastructures of access networks are underutilized during the nighttime [25]. What the SRC and SDR soft base station system need to do is to provide the proper amount of resources according to its historic statistics to support users’ demands over time.

However, this centralized way of allocating radio resources clearly cannot support the demand–supply balance well for different base stations. One intrinsic feature of the future 5G network is allowing each terminal user to select his access point freely to maximize his personal experience. For a given radio base station, its terminal users may join or leave in an unpredictable manner, which may lead to frequent underloading or overloading of the base station. Therefore, one key question in future 5G networks is how terminal users should adaptively select their radio access points in a rational manner, while maximizing the network-level performance as much as possible simultaneously?

## 3. Methods

### 3.1. Network Selection Problem Definition

Based on the aforementioned dynamic HetNet environment, we make the following assumptions: (1) The provided bandwidth of each base station is varying over time; (2) Each user can send a connection request to at most one base station at a time; (3) Each user can only have access to the state information of the base station it connected to from completed interactions and lacks prior knowledge of any other networks or terminal users. The cooperation between the user and its connected base station is helpful and doesn’t infringe upon any other’s interest.

#### 3.1.1. Multiagent Network Selection Model

In practice, each user makes independent decisions based on its local information without communication with others. However, actions taken by users influence the actions of others indirectly. Therefore, we formulate the problem as a multiagent coordination model [26], in which a population of terminal users located in the same or different service areas with no information about others learn to compete to maximize their payoffs given that the available bandwidth varies dynamically. Formally, the multiagent network selection scenario is modeled as a 6-tuple $<BS,B_{k}\left(t\right),U,b_{i}\left(t\right),A_{i},P_{i}\left(t,a\right)>$, where:

- BS={1,2,…,m} is the set of available base stations (BSs) in the HetNet environment.
- $B_{k}\left(t\right)$ denotes the provided bandwidth of base station k∈BS at time *t*, which varies over time.
- U={1,2,…,n} is the set of terminal users involved.
- $b_{i}\left(t\right)$ denotes the bandwidth demand of user i∈U at time *t*, which also changes over time.
- $A_{i}\subseteq BS$ is the finite set of actions available to user i∈U, and $a_{i}\in A_{i}$ denotes the action (i.e., selected base station) taken by user *i*.
- $P_{i}\left(t,a\right)$ denotes the expected payoff of user i∈U by performing the strategy profile $a=\left\{a_{1},\ldots ,a_{i},\ldots a_{n}\right\}\in \times_{j\in U}A_{j}$ at time *t*.

There are *n* users competing for *m* base stations in the system. The detailed definition of instantaneous payoff of user *i* based on the joint strategy profile a can be expressed as

(1) $$P_{i}\left(t,a\right)=\alpha \log\left(1+\beta \frac{w_{i}\left(t,a\right)}{b_{i}\left(t\right)}\right),$$

where α and β are constants that control the shape of the function, and $w_{i}\left(t,a\right)$ is the perceived bandwidth of user *i* at time *t*. Here, perceived bandwidth is a theoretical value that does not consider the transmission loss. The payoff function monotonically increases as the perceived bandwidth increases. In this payoff function, we mainly consider the user’s bandwidth demand and the perceived bandwidth, which are the two most rudimentary factors for users. The proposed formulation indicates the relationship between the perceived bandwidth and the bandwidth demand, i.e., the bandwidth satisfaction for a user. It is applicable to many applications on the Internet (e.g., elastic services like file transfer and web browsing using transmission control protocol) [27].

#### 3.1.2. Theoretical Analysis

We identify two major properties underlying the multiagent network selection problem. Nash equilibrium (NE) is the most commonly adopted solution concept in game theory. Under an NE, no player can benefit by unilaterally deviating from its current strategy [4]. Underlying the multiagent network selection problem, an NE is reached when there is no overload on any base station (this situation is shown in later experiments). Under this condition, users’ perceived bandwidth equals their demands and all users’ payoffs reach a maximum. Therefore, no one is willing to change his strategy given that others’ strategies are unchanged.

**Definition** **1.** $a^{*}\in \times_{i\in U}A_{i}$ *is a Nash equilibrium if for all* $k\in BS,\sum_{j}b_{j}\left(t\right)\leq B_{k}\left(t\right)$, *where* $j\in \{j\in U|a_{j}=k,a_{j}\in a^{*}\}$.

However, an NE may not be desirable in general since it may not necessarily correspond to the maximization of the system-level payoff. Fortunately, any NE in our model is also Pareto optimal and socially optimal [4]. The two properties guarantee both the system’s stability and system-level optimization.

**Theorem** **1.** *Nash equilibrium, Pareto optimality and Social optimality are equivalent in the multiagent network selection problem*.

**Proof.**  It can be deduced that, if profile $a^{*}$ is an NE, each user’s payoff reaches a maximum and cannot be further increased. Therefore, it is impossible to find another outcome under which no user’s payoff is decreased while at least one user’s payoff is strictly increased. This proves that $a^{*}$ is Pareto optimal. In addition, $P_{i}\left(t,a^{*}\right)=\max P_{i}\left(t,a\right)\Rightarrow \sum_{i}P_{i}\left(t,a^{*}\right)=\max\sum_{i}P_{i}\left(t,a\right),\forall a\in \times_{j\in U}A_{j}$. The sum of all users’ payoffs reaching a maximum means $a^{*}$ is also socially optimal. ☐

### 3.2. Multiagent Network Selection Strategy

The adaptive network selection strategy described in Algorithm 1 is integrated in each terminal user. Each user only communicates with its currently connected base station and has no communication with others. Any user is allowed to join or leave the environment and trigger a call request at any time. The prior information before an initial selection available to user i∈U is its own bandwidth demand $b_{i}$ and the available base station set BS.

For each user, its strategy consists of two steps: selection (Line 2) and evaluation (Line 4). In selection procedure, the user learns to choose the best candidate network to satisfy its special demand. Once the selection procedure is completed, the user gets the feedback of a 2-tuple <load,bandwidth> from its connected base station in the last interaction as a historical record. After that, an evaluation procedure will be triggered to update the strategy. More details about selection and evaluation are described in Section 3.2.1 and Section 3.2.2, respectively.

**Algorithm 1** Network selection algorithm for each user**Input:** available base station set BS    bandwidth demand $b_{i}$**Output:** selected base station seleBS1: **loop**2:  seleBS← Selection()3:  receive the feedback of state information in the last compelted interaction4:  Evaluation()5: **end loop**

#### 3.2.1. Selection

To make an informed selection decision, a user may need the state information on each base station, which, however, is either with high communication costs or unavailable beforehand. To address this issue, our approach is based on different users’ beliefs, represented by diverse predictors and historic information about the environment, in order to forecast the possible load and bandwidth for further decisions.

Algorithm 2 summarizes the selection procedure for user i∈U. For each available base station k∈BS, the user checks whether it can satisfy its special demand (Lines 1–11). If the user sends a connection request to a base station with no historic information, which is the standard case at the beginning of the life-cycle, this unpredictable base station will be added in a spare list for a later decision (Lines 2–3). Otherwise, the user predicts provided bandwidth and possible load of each base station (Lines 5–6). If the predicted load plus the demand is below the predicted bandwidth, this base station is added to the list of candidates (Lines 7–8).

Then, the user evaluates if any candidate base station is expected. There might be three cases. In the case where the list of candidate base stations predicted having adequate bandwidth available is not empty (Line 12), the “best network selection” is determined by the following policy: the base station with the most expected free bandwidth is chosen as the most appropriate connection currently (Line 16). In particular, in the case there is no available candidate, the user will randomly explore one from all unpredictable base stations to gather its state information (Lines 17–18). There also might be an exceptional case that no base station is generated from the algorithm (i.e., no base station could satisfy the user’s demand) (Line 19). In this case, the original base station is used and flag is set into −1.

Each user maintains a historic information table $table_{k}$ for each connected base station *k*. The table is composed of up to *m* items $h_{j}=\left(t_{j},load_{j},bw_{j}\right)$, comprising observed time $t_{j}$, observed load $load_{j}$ and observed bandwidth $bw_{j}$. The oldest item will be rewritten if *m* items are already recorded because dynamic environments require more up-to-date information to make more reliable predictions. Formally, $table_{k}$ is expressed as the following equation:

(2) $$\begin{array}{c} table_{k}=\left(h_{0},\ldots ,h_{p}\right)=\\ \left(\left(t_{0},load_{0},bw_{0}\right),\ldots ,\left(t_{p},load_{p},bw_{p}\right)\right),\left(0\leq p<m\right). \end{array}$$

**Algorithm 2** Selection1:**for all**
k∈BS
**do**2: **if**
$table_{k}=⌀$
**then**3:  push *k* in unpredList4: **else**5:  predLoad←LoadPredict($p^{A}$)//
$p^{A}$ active predictor6:  predBW←BWPredict()7:  **if**
$predLoad+b_{i}\leq predBW$
**then**8:   push *k* in candList9:  **end if**10: **end if**11:**end for**12:**if**
candList≠⌀
**then**13: **for all**
cand∈candList
**do**14:  availBW=predBW−predLoad15: **end for**16: $seleBS\leftarrow {\mathrm{argmax}}_{k\in BS}\left(availBW\right)$17:**else if**
unpredList≠⌀
**then**18: seleBS←random(unpredList)19:**else**20: seleBS←lastBS// stay at last BS21: flag=−122:**end if**

The load prediction mechanism employs time series forecasting techniques to predict future load value based on previously observed values. It involves three major steps:

1 Create predictor set. Each user keeps a set of *r* predictors $P\left(a,k\right)=\left\{p_{i}|1\leq i\leq r\right\}$, which is created from some predefined set in evaluation procedure (Section 3.2.2, case 1), for each available base station *k*. Each predictor is a function from a time series of historic loads to a predictive load value, i.e., $f:(\left(t_{i},load_{i}\right)|i=0,‥,p)\to predLoad$.
2 Select active predictor. One predictor $p^{A}\in P$ is called active predictor, which is chosen in the evaluation procedure (Section 3.2.2, case 2,3), used in real load prediction.
3 Make forecast. Predict the base station’s possible load via its historic load records and the active predictor.

A similar prediction mechanism can also be adopted to bandwidth prediction.

#### 3.2.2. Evaluation

After the user’s selection has finished, the evaluation procedure introduced in Algorithm 3 is performed. This process is divided into three cases based on the selected base station.

**Algorithm 3** Evaluation1:**if**
predictorSet=⌀
**then**2: create predictorSet for seleBS3: $p^{A}\leftarrow$random(predictordSet)4: update$\left(table_{seleBS}\right)$5:**else if**
flag=−1
**then**6: **for all**
k∈BS
**do**7:  delete $h\in table_{seleBS}$ with a probability8: **end for**9:**else**10: **for all**
p∈predictorSet
**do**11:  predLoad←LoadPredict(p)12:  $r_{p}=1-\frac{\left|load-predload\right|}{load}$13:  $Q_{p}=\left(1-\alpha \right)Q_{p}+\alpha r_{p}$14: **end for**15: $p^{A}\leftarrow$BoltzmanExploration(predictordSet)16: // abruptly changing environment17: **if**
$\left|B_{seleBS}-predBW\right|>\Delta$
**then**18:  $d=\left|B-lastBW\right|$19:  **for all**
$h\in table_{seleBS}$
**do**20:   h←h±d21:  **end for**22: **end if**23: update$\left(table_{seleBS}\right)$24:**end if**

**Case 1** If the selected base station is visited for the first time, the user will create a new predictor set for this base station and record its state information into the corresponding record table (Lines 1–4). All predictors in the set are chosen randomly from a predefined set, hence users’ predictor sets may be different from each other. As displayed in Table 1, the predefined set contains multiple types of forecasting functions [28] differ in window sizes. Different types of predictors are suitable for different situations and environments.

**Case 2** If flag=−1, it implies that currently historical records recommended no appropriate base station (Line 5). In this case, some old records need to be removed from the table to get more up-to-date information for further predictions (Line 6–8), which is necessary for a successful adaptation in the future. Otherwise, the user will never get an opportunity to access other base stations, which may satisfy its demand very well.

**Figure 3:**
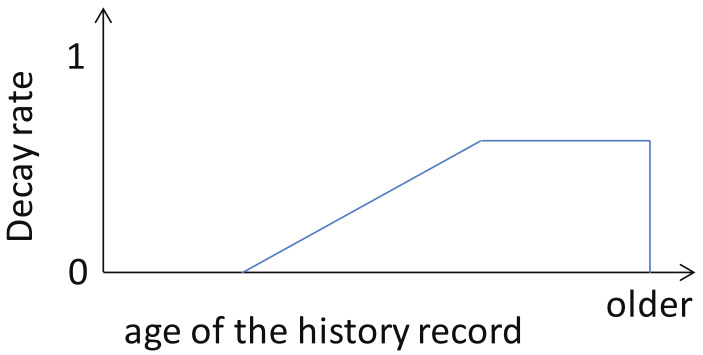
An example of decay rate of the historical record.

Old historical records are removed using a decay rate. The decay rate is a cumulative distribution function used to calculate the probability that a historical record is deleted after it has reached a certain age. An example of such a cumulative distribution function is given in Figure 3. A relatively newer record has a lower deleting probability; otherwise, an older one has a higher deleting probability. However, depending on the environment, the probability density function must be altered. If the number of base station candidates per user is high, historical information must be kept longer to avoid the exploration of unexplored base stations and reduce the switching rate.

**Case 3** The general situation is that the user switched to a previously visited base station, i.e., it already has historical records on this base station (Line 9). The evaluation mainly involves two aspects: assessing the performance of all predictors in the set (Line 10–15) and dealing with the case of abruptly changing bandwidth (Line 17–22). The assessment of predictors resorts to *Q*-learning. Specifically, the *Q*-function in our approach is defined as the following equation:

(3) $$Q_{p}\left(t\right)=\left(1-\alpha \right)Q_{p}\left(t-1\right)+\alpha r_{p}\left(t-1\right),$$

(4) $$r_{p}=1-\frac{\left|load-predload\right|}{load},$$

where p∈predictorSet denotes the predictor, $Q_{p}\left(t\right)$ is the *Q*-value of *p*, and α is the learning rate. The prediction accuracy, which is the error of the prediction compared to the observed value, is taken into consideration. We use the observed reward $r_{p}$ to denote the prediction accuracy of *p*. The predictor that forecasted a more exact value receives a higher reward; otherwise, it receives a lower reward.

In our approach, the Boltzman exploration mechanism [29] is adapted to explore the active predictor (Line 15). The probability $x_{p}$ of selecting predictor *p* is given by

(5) $$x_{p}\left(t\right)=\frac{e^{Q_{p}\left(t\right)/T}}{\sum_{k}e^{Q_{k}\left(t\right)/T}},$$

where the temperature T>0 balances the tradeoff between exploration and exploitation: when T→0, the user plays a Greedy policy in which the predictor with the maximum *Q*-valve is selected (pure exploitation), whereas, for T→∞, user’s selection is completely random (pure exploration). Since $x_{p}\left(t\right)$ is the increasing function of *Q*-valve, the predictor with higher prediction accuracy is chosen with a higher probability.

The above process works well in the environment with gradually changing bandwidth. However, it is slightly different in an abruptly changing case. When detecting that the difference between the observed bandwidth and predicted bandwidth of the base station is larger than a threshold Δ, the user will consider that it encounters an abruptly changing environment (Line 17). At catastrophe points, all historical records are invalid and may lead to inaccurate predictions. In order to eliminate the adverse influence and achieve rapidly re-converging, records in the table are revised to be new references. If there is a sudden rise of bandwidth, the difference *d* is added on each old record; otherwise, *d* is subtracted from each old record.

This algorithm is based on the multiagent network selection model that may be influenced by the parameter of the number of agents. Thus, the terminal user number has a great influence on the complexity of the algorithm, i.e., the convergence time may increase with the increasing user number.

## 4. Results

In this section, we first present the simulation results of our approach in terms of adaptability, user payoff, switching rate, bandwidth utilization and convergence time under various user numbers in both gradually and abruptly changing environments. After that, we experimentally compare the performance of our approach with two existing multi-user network selection algorithms ([9,17]). Finally, the robustness of our approach is examined.

Parameter settings of the simulated scenario are given in Table 2. We consider a variety of HetNet environments consisting of up to 800 users, which involve the following three aspects. All experimental results are averaged over 50 independent runs.

- RAT type: we consider three typical networks with various radio access technologies (RATs), namely IEEE 802.11 Wireless Local Area Networks (WLAN), IEEE 802.16 Wireless Metropolitan Area Networks (WMAN) and OFDMA Cellular Network, which are represented by $BS_{i}\left(i=0,1,2\right)$. Multi-mode user equipment in the heterogeneous wireless network can access any of the three networks.
- provided bandwidth: the maximum provided bandwidth of the three networks are 25 Mbps, 50 Mbps, and 5 Mbps, respectively [30]. Without loss of generality, two types of changing environments based on historical statistic traffic are considered. One of them is simulated as sinusoidal profiles, which change gradually. The provided bandwidth may also change abruptly according to time division, such as dawn, daytime and evening.
- bandwidth demand: users’ bandwidth demands also vary in a reasonable range. There are two types of traffic demand in the area: real-time voice traffic and non-real-time data traffic, which are randomly distributed.

For the payoff function (Equation (1)), we set α=1 and β=1.7, so that, when perceived bandwidth equals the demand, the payoff reaches a maximum value of 1. Here, a proportional bandwidth allocation mechanism [31] is employed, which can be simply presented as

(6) $$w_{i}\left(t,a\right)=\left\{\begin{array}{c} b_{i}\left(t\right),\;\sum_{j}b_{j}\left(t\right)\leq B_{a_{i}}\left(t\right),\\ \frac{B_{a_{i}}\left(t\right)\cdot b_{i}\left(t\right)}{\sum_{j}b_{j}\left(t\right)},\;otherwise, \end{array}\right.$$

where $j\in \{j\in U|a_{j}=a_{i},a_{j},a_{i}\in a\}$ is the user who takes the same action $a_{i}$ (i.e., connects the same base station) with user *i*. Here, the perceived bandwidth is a theoretical value that does not consider the transmission loss.

### 4.1. Experiment Results

This section studies the impact of the number of users on system performances from the following aspects. Two demand situations are simulated: (1) the total amount of bandwidth demand is close to, but less than the provided bandwidth of all base stations; (2) the total amount of bandwidth demand is beyond the total provided bandwidth.

**Figure 4:**
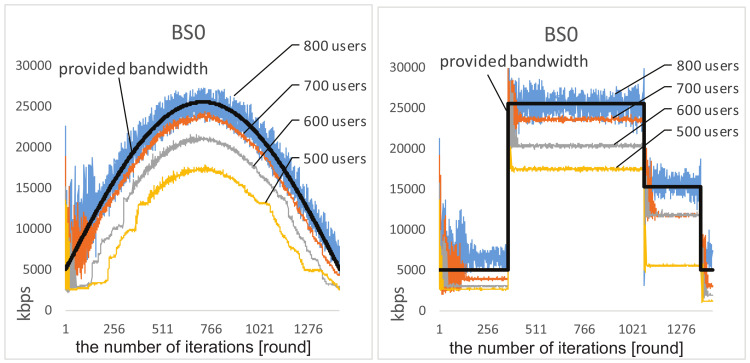
Load situations on $BS_{0}$ under various user numbers.

**Adaptability**. Figure 4 shows the behavior of network selection on $BS_{0}$ within gradually and abruptly changing environments.When the user number increases from 600 to 700, the total demand is less than the total provided bandwidth. Initially, all users randomly select their base stations, thus resulting in high levels of overload or underload on different base stations. However, after a short period of interactions, all users can learn to coordinate their selections and the network bandwidth of $BS_{0}$ becomes well-utilized without being overloaded. Moreover, we observe that the increase of user number leads to better adaptability. Intuitively, this indicates that, when the total demand reaches close to the upper bound of the provided bandwidth, users can more sensitively sense the dynamic environment and quickly accommodate to the changes.

When there are 800 users involved, the total demand exceeds the provided bandwidth, it can be seen that, after a period of interactions, the amplitude of the load decreases significantly. A similar phenomenon can be observed on $BS_{1}$ and $BS_{2}$.

**Figure 5:**
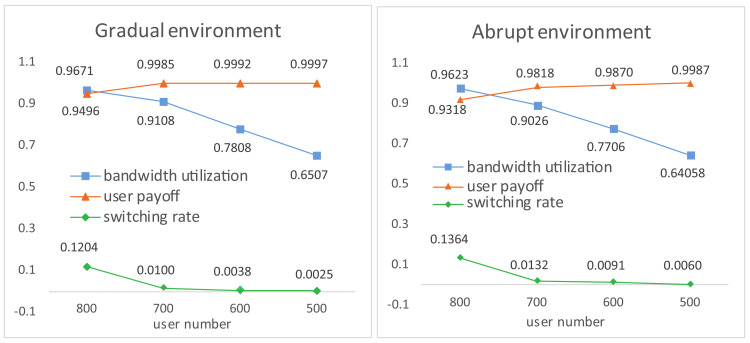
User payoff, switching rate and bandwidth utilization under various user numbers.

**User Payoff, Switching Rate and Bandwidth Utilization**. In Figure 5, increasing user number results in a slight decrease in user payoff while a marginal increase in switching rate. With increasing competition of limited base stations and bandwidth, the average bandwidth utilization efficiency increases approximately linearly. The three performances are a little worse in abruptly changing environments due to jitters at catastrophe points.

**Figure 6:**
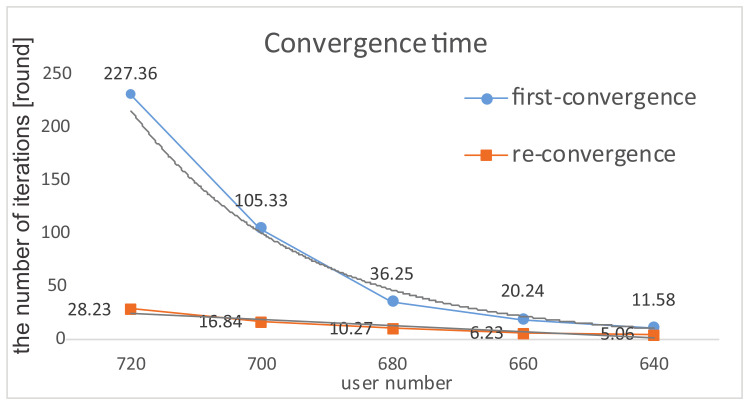
Average convergence time.

**Convergence Time**. The terminal user number plays a great influence on the complexity of our proposed algorithm (i.e., the convergence time). When the total demand is less than the total provided bandwidth, the system guarantees convergence, i.e., if there is no overload on any base station, the system converges to Nash Equilibrium, which is also Pareto optimal and socially optimal (Definition 1, Theorem 1). Initially, the system takes a learning phase to achieve convergence. If the provided bandwidth changes gradually or stays static, the equilibrium is sustained over time. We call it first-convergence, and the average first-convergence time exponentially increases in an acceptable range with the increasing user number from 640 to 720. Especially, in an abruptly changing environment, when encountering catastrophe points, the equilibrium is broken but re-converges in a number of steps. The average re-convergence time linearly varies with the number of users (see Figure 6).

### 4.2. Experiment Comparisons

In this section, we compare our adaptive learning based algorithm (ALA) with two existing multi-user network selection algorithms in both gradually and abruptly changing environments. The first one is a classical Q-learning based algorithm (QLA) [9]. In QLA, each user maintains a Q-value for each available network, which is learned knowledge about the network. The user selects the network with maximal Q-value. The second one is RAT selection algorithm (RATSA) [17]. RATSA is similar with the best response, where each user always selects the network with maximal expected throughput.

**Communication Complexity**. We first compare the communication complexity of the three algorithms in Table 3. Before an initial selection, each user has the knowledge of its base station candidates and bandwidth demand. In ALA, we assume that there exists a cooperation between a user and it connected base station. The user gets the feedback of a tuple <load,bandwidth> from the base station in previous connections, rather than any prior knowledge. Such a cooperation is available and helpful, but does not infringe upon interests of any others.

RATSA requires a significant amount of global information, such as the provided bandwidth of each base station at the next time, the number of users in each base station, and the number of past consecutive migrations on the selected base station. However, in a real network scenario, it is impossible for a user to get this future information. Therefore, ALA based on learned and predicted information is more practically feasible and effective. In QLA, users making decisions only depends on their local information (i.e., Q-values which are updated by their rewards) with lower communication complexity. However, it turns out to give bad performances under a changing environment presented in later experiments.

**Figure 7:**
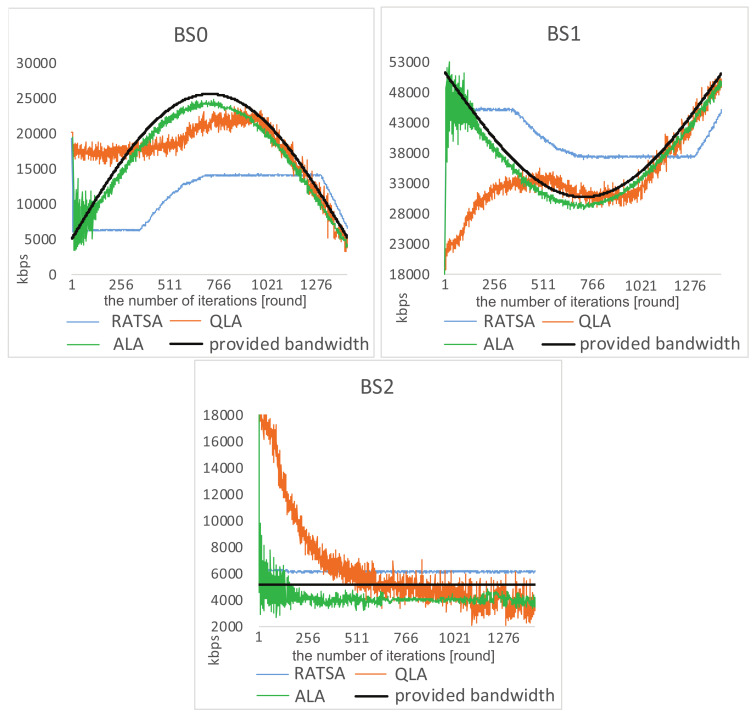
Comparisons of load situations in a gradually changing environment.

**Figure 8:**
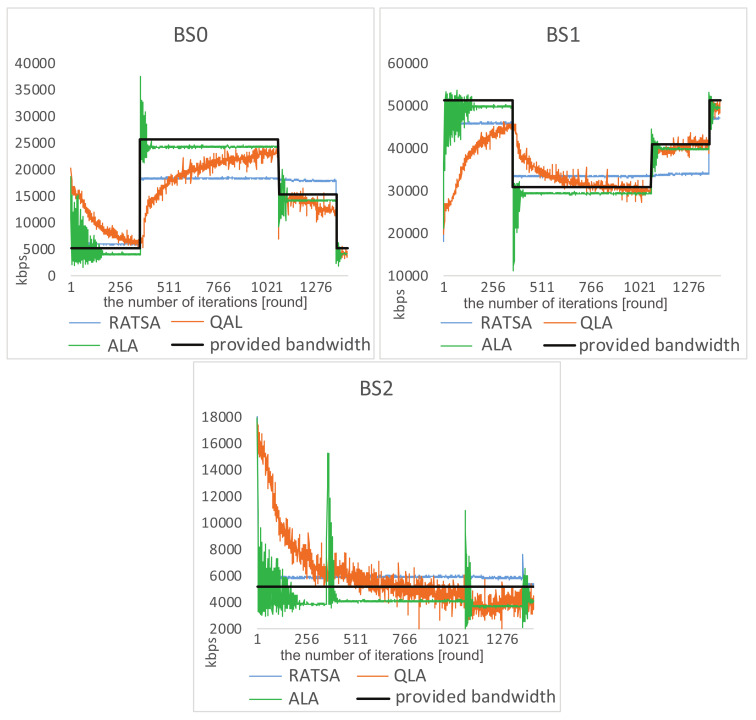
Comparisons of load situations in an abruptly changing environment.

**Load Balancing Analysis**. We investigate the load situations on the three base stations for some time when there are 720 users involved (see Figure 7 and Figure 8). It is the case that the total bandwidth demand is quite close to the total amount of provided bandwidth.

We observe that, under ALA, after a few learning steps, there is no overload, and the load on each base station dynamically changes with the amount of provided bandwidth. This implies that the system converges to equilibrium and achieves load balancing among the three base stations. It is worth noting that the jitter on $BS_{2}$ in Figure 8 is because users are trying to join or leave this base station in response to the abrupt changes on the other two base stations.

In RATSA, a user switches its base station only if the value of allocated bandwidth from other base station divided by currently perceived bandwidth is higher than a given threshold η. Note that only one user is allowed to make the switch each time. The threshold η can greatly impact the system performance. For fair comparisons, we set η=1.5 in the following comparisons, which gives RATSA the best performance. The comparative figures show an unbalancing phenomena that there is too much unmet demand on $BS_{1}$ and $BS_{2}$, but too little utilization on $BS_{0}$ over some time. This indicates that users cannot sense the dynamic environment and adjust their strategies timely.

We also simulate the network selection scenario using another learning based approach. In QLA, it can be observed that users are trying to adapt to the changing environment. However, it takes a long time to get close to the varying bandwidth and complete load balancing cannot be achieved. Moreover, when getting to catastrophe points, users have to relearn to adapt to the new environment.

**Figure 9:**
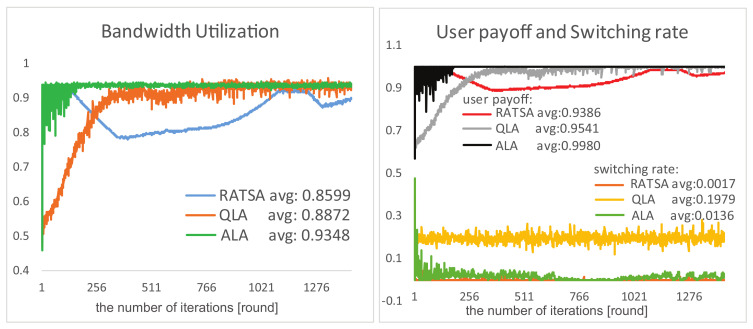
Comparisons of performances in a gradually changing environment.

**Figure 10:**
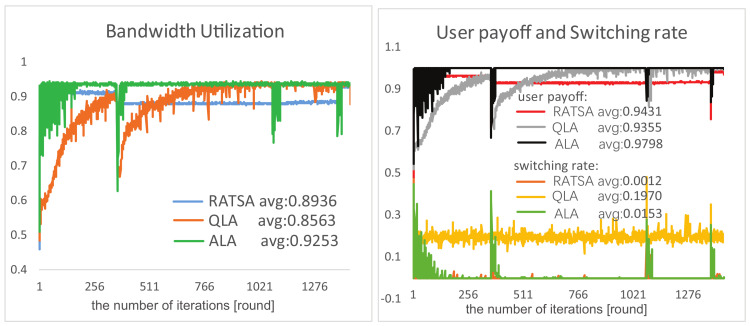
Comparisons of performances in an abruptly changing environment.

**User Payoff, Switching Rate and Bandwidth Utilization**. Comparison results of the three algorithms in terms of user payoff, switching rate and bandwidth utilization are presented in Figure 9 and Figure 10 under two changing environments, respectively. We observe that, in the beginning period, RATSA performs better than ALA in bandwidth utilization and user payoff; however, ALA outperforms it after a few interactions and shows better performance thereafter. The switching rate of ALA is slightly higher because users try to switch their connections to respond to the dynamics to get higher payoffs in the initial phase and at catastrophe points. It is important to highlight that the jitters in the abruptly changing environment of ALA are because of the time-lag of detecting abruptly changing bandwidth. This phenomenon may not exist in RATSA, since users are assumed to always access the currently and next provided bandwidth of all base stations, which is usually not accessible in practical environments.

As for QLA, although we can sense it is trying hard to adapt to the dynamic environments, it demonstrates bad performances of any of the three criteria compared with ALA, especially switching rate.

### 4.3. Robustness Testing

It has been assumed so far that all terminal users have adopted the adaptive learning based network selection approach (i.e., ALA) in previous simulations. In this section, the experimental study for ALA examines the robustness of the approach when this assumption is violated. In practise, 100% compliance by users is unlikely to be achieved [32]. This is because some users may not be convinced to participate or, even if they are all willing to participate, some of them may come across different problems such as information and sensing limitations.

**Figure 11:**
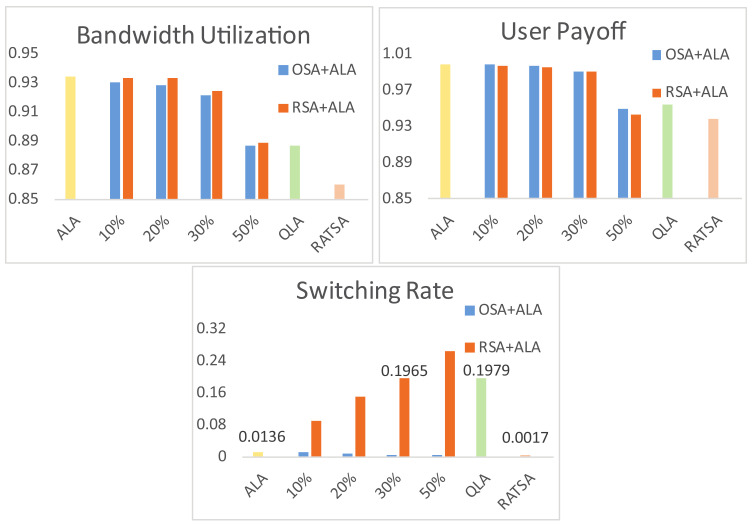
Robustness of the adaptive and learning based network selection approach.

Figure 11 depicts the system performance in the presence of 10%, 20%, 30% and 50% non-compliant users when the environment changes gradually (it shows a similar phenomenon when the environment changes abruptly, which is omitted due to the space limitation) with 720 terminal users involved in total. Non-compliant users are simulated as agents sticking to their initial choice (OSA) or keeping a random selection approach (RSA). It is observed that with 10% to 30% non-compliant users, bandwidth utilization and user payoff are not affected much and, as a result, the system using ALA still outperforms the system using the other two approaches (i.e., QLA and RATSA). The two performances are almost equal to QLA or RATSA when there are 50% non-compliant users participating.

As for switching rate, when non-compliant users takes OSA, this performance remains desirable. When the non-compliant users take RSA, the switching rate increases linearly with the number of non-compliant users because of the random selection of these users. However, within 30% non-compliant users, the switching rate is still lower than the performance when users use QLA. The results suggest that ALA works well under non-compliant users within 50% and the system demonstrates a good robust performance.

## 5. Discussion

From Section 4, extensive simulation results show that our approach achieves significantly better performance compared with two learning and non-learning based approaches in terms of load balancing, user payoff, switching rate, the overall bandwidth utilization efficiency, etc. It is also robust against failures of terminal users: when they occasionally join or leave, the system can self-organize quickly and adapt to a newly created environment. Our approach overcomes the defect of requiring too much state information, and can handle the unpredictable dynamics of wireless network resources. It can be well applicable for dynamic 5G network environments to make heterogeneous network selection decisions.

The idea of the paper is inspired by some learning algorithms in literatures [33,34]. However the application in wireless network environments has some shortcomings. Even though the switching rate has been reduced to a relatively low level, there still exist some ping-pong effects due to the fact that terminal users may switch among base stations when unnecessary. Therefore, one interesting direction is to investigate eliminating the ping-pong effect and better improving QoS of users. Unfortunately, the performance of power consumption cannot be simulated due to limited experiment conditions, but it should be investigated in the next work. Additionally, future work should take into account more complicating factors such as distance and mobility.

## 6. Conclusions

In this paper, an adaptive learning based approach is presented to tackle the network selection problem with changing bandwidth in HetNet environments. We investigate the performance of the algorithm under various conditions and parameters: the bandwidth of networks changes both gradually and abruptly, the number of terminal users increases from less to more, and the requirements of users also vary over time. The simulation results demonstrate that our approach enables a population of terminal users to adapt effectively to the dynamics, which, on the whole, results in little overload or underload. The system ideally converges to Nash equilibrium when there is no overload on any base station, which is also Pareto optimal and socially optimal. Moreover, we compare our results with two existing approaches and get significantly better performances. Finally, the robustness of our proposed approach is examined, for which the system keeps desirable performances with up to 50% non-compliant terminal users.

## Figures and Tables

**Table 1:** Time series forecasting methods.

Method	Description (Window Size x≤p)
Weighted Average	$predload=\sum_{i=0}^{x}w_{i}load_{i}$ $\sum_{i=0}^{x}w_{i}=1$
Geometric Average	$predload=\sqrt[{x+1}]{\prod_{i=0}^{x}load_{i}}$
Linear Regression	$predload=\hat{a}t+\hat{b}$ ($\hat{a},\hat{b}$ can be obtained by using least square method)
Exponential Smoothing	$predload_{t+1}=\alpha load_{t}$ $+\left(1-\alpha \right)predload_{t}$ $=\sum_{i=0}^{x}\alpha {\left(1-\alpha \right)}^{i}load_{t-i}$

**Table 2:** Parameter settings.

Access Tech	Network Rep	Base Station	Maximum Bandwidth	User Demand
WLAN	Wi-Fi	$BS_{0}$	25 Mbps	voice traffic: 32 kbps
WMAN	WiMAX	$BS_{1}$	50 Mbps	data traffic: 64
OFDMA Cellular Network	4G	$BS_{2}$	5 Mbps	kbps ∼ 128 kbps

**Table 3:** Comparisons of communication complexity.

Algorithm	ALA	RATSA	QLA
common information required	before selection: BS candidates; bandwidth demand. after selection: perceived bandwidth *w* from selected BS.		
different information required	1. previous provided bandw-idth of selected BS. 2. histroical load on selected BS.	1. future provided bandwidth of each BS. 2. number of users on each BS. 3. number of past consecutive migrations on selected BS.	–
base stations to be communicated	selected BS	all BS candidates	selected BS
influencing parameter	–	switching threshold η	–
